# Genetic profiling of primary and secondary tumors from patients with lung adenocarcinoma and bone metastases reveals targeted therapy options

**DOI:** 10.1186/s10020-020-00197-9

**Published:** 2020-09-17

**Authors:** Long Huang, Xiao-Liu Jiang, Hong-Bin Liang, Jian-Cheng Li, Li-Han Chin, Jian-Ping Wei, Rui-Ru Wang, Jing Cai, Qiang Xiong, Lien-Tu Wang, David S. Cram, An-Wen Liu

**Affiliations:** 1 Department of Oncology, the Second Affiliated Hospital of Nanchang University, Nanchang, China; 2 JiangXi Key Laboratory of Clinical and Translational Cancer Research, Nanchang, China; 3 Berry Oncology Corporation, Beijing, China; 4 Berry Genomics Corporation, Beijing, China; 5 Department of Oncology, Fujian Cancer Hospital & Fujian Medical University Cancer Hospital, Fuzhou, China

**Keywords:** Lung adenocarcinoma (LA), Lung adenocarcinoma bone metastasis (LABM), Epithelial growth factor receptor (*EGFR*), clonal evolution, capture single molecule amplification and resequencing technology (capSMART)

## Abstract

**Background:**

Patients newly diagnosed with lung adenocarcinoma with bone metastases (LABM) have poor survival rates after treatment with conventional therapies. To improve outcomes, we retrospectively investigated whether the application of a more comprehensive genetic test of tumor biopsies samples from LABM patients could provide the basis for treatment with more effective tyrosine kinase inhibitors (TKIs) regimens.

**Methods:**

Fine needle biopsies were taken from the primary tumor (PT) and a secondary bone metastasis (BM) of 17 LABM patients before treatment. Simple genetic profiles for selecting therapies were initially obtained using an ARMS-PCR test for *EGFR* and *ALK* fusion mutations. More detailed genetic profiles of somatic exon SNVs and CNVs in 457 cancer-related genes were retrospectively derived using capture single molecule amplification and resequencing technology (capSMART).

**Results:**

ARMS-PCR identified 14 *EGFR* positive, 3 *EGFR* negative and 1 *ALK* fusion positive patient. A therapy regimen incorporating TKIs Gefitinib and Crizotinib was offered to the *EGFR* and *ALK* fusion positive patients, respectively. With the exception of two patients, molecular profiling of matching PT and BM biopsies identified a highly shared somatic variant fingerprint, although the BMs exhibited additional genomic instability. In six of 13 *EGFR* positive patients and in all three *EGFR* negative patients, examination of the genetic profiles identified additional clinically significant mutations that are known or experimental drug targets for treatment of lung cancer.

**Conclusion:**

Our findings firstly suggest that treatment regimens based on comprehensive genetic assessment of newly diagnosed LABM patients should target both the PT and secondary BMs, including rogue clones with potential to form new BMs. Second, the additional information gained should allow clinicians to design and implement more personalized treatment regimens and potentially improve outcomes for LABM patients.

## Background

Lung cancer is one of the most common types of cancer worldwide with an estimated 1.8 million new cases diagnosed annually (Ferlay et al. 2015). The vast majority of all cases (85%) are non-small cell lung cancer (NSCLC), with lung adenocarcinoma (LA) being the most common pathological subtype (Travis 2011). In China, lung cancer ranks first for morbidity and mortality (Chen et al. 2016) with approximately 30–40% patients presenting with metastatic disease. Many of these patients who have initial curative therapies eventually relapse (Ettinger et al. 2008; Yu et al. 2011). The 5-year survival rate of lung cancer patients treated with standard therapies is around 20% (Bender 2014).

In LA patients, the most common tissues where metastases form are the liver, brain and bone (Hess et al. 2006; Stenbygaard et al. 1999; Tas et al. 1999) and those with bone metastases (LABM) generally have a poorer prognosis since they are often diagnosed late in the course of the disease. Once bone metastasis occurs, around 80% of patients will suffer constant pain with a much-decreased quality of life (Chow et al. 2009; Decroisette et al. 2011; Harris et al. 2009; Tsuya et al. 2007). Surgical treatment, radiotherapy, chemotherapy and targeted therapy comprise the treatment regimens available to treat LABM patients but even after clinical treatment, the median survival time is still only 6–10 months and, at 1 year, the survival rate is only 40–50% (Bender 2014; Hess et al. 2006; Tas et al. 1999).

Genetic profiling of primary tumor biopsy samples from LA patients at different TNM stages has identified specific driver mutations in a number of genes, including *EGFR*, *ALK*, *BRAF*, *KRAS* and *TP53* (Inamura 2018; Sharma et al. 2007; Wang et al. 2017; Zhang et al. 2016). Up to 50% of LA patients have *EGFR* mutations, the most common being in-frame exon 19 deletions and a missense mutation L858R, that cause tyrosine kinase (TK) domain hyperactivity which then drives tumorogenesis (Khalil and Altiok 2015). *EGFR* mutation positive patients can often be treated successfully with new generation tyrosine kinase inhibitors (TKIs) that are effective in the down-regulation of TK activity (Gridelli et al. 2011; Holleman et al. 2019). Compared to LA patients with wild-type *EGFR*, patients with *EGFR* driver mutations are at a higher risk for development of bone metastasis (Bi et al. 2018), indicating the importance of early diagnosis and treatment.

**Fig. 1:**
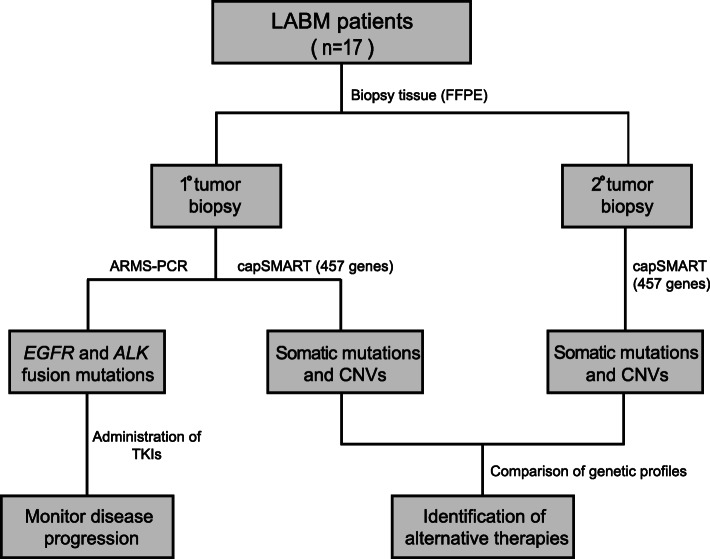
Study design

Currently, for LABM patients, there is a paucity of information on the key genetic changes present in the primary tumor and clonal variants that manifest as metastases. In several studies of naïve untreated patients with different tumor types, including lung tumors, detailed genetic profiling of the primary tumors and secondary metastases showed that while the clonal metastases closely mimicked the genetic changes in the primary tumor, new driver and passenger oncogenic mutations as well as copy number variations (CNVs) can arise (Hu et al. 2019; Reiter et al. 2018). This suggests that a more comprehensive knowledge of the genetic changes in tumors of individual LABM patients could provide a sounder approach for more effective therapy based on the genetics, that will not only target the primary tumor, but also the breakaway metastasis clones. To test this hypothesis, our study first compared the mutation and CNV profiles of primary and metastasis tissue samples in 17 LABM patients after screening 457 cancer associated genes for somatic variants (Fig. 1). We then assessed whether this strategy could reveal personalized targeted therapy options effective against both the primary tumor and secondary metastases.

## Methods

### Patients

The clinical research study was approved by the Local Ethics Committee IRB of The Second Affiliated Hospital of Nanchang University (Number 2017–008). Between January 2017 to December 2018, 17 patients diagnosed with LABM were recruited to the study (Table 1, Supplementary Table 1). Patients provided written informed consent for collection of tumor biopsy samples and genetic profiling to identify individual targeted therapies (Fig. 1).

**Table 1:** Tumor characteristics and treatment outcomes

Patient	Age (Sex)	Tumor size (cm)		TNM stage	Tumor histology		ARMS-PCR		Tumor purity^a^		Treatment	Disease outcome^c^ (months)	
		1°	2°		1°	2°	1°	2°	1°	2°		1°	2°
P1	50 (F)	1.8 × 2.0	1.6 × 1.7	cT3N2M1b IVa	AD	MAD	E19 del	NT	55%	51%	Gefitinib + RT	Progressive (8)	Progressive (8)
P2	70 (M)	2.3 × 1.7	3.3 × 2.5	cT4N0M1b IVa	AD	MAD	E19 del	NT	31%	20%	Gefitinib + RT	Stable (18)	Stable (18)
P3	54 (M)	2.3 × 1.7	3.0 × 2.0	cT1N2M1b IVa	AD	MAD	E19 del	NT	50%	45%	Gefitinib + RT	Stable (10)	Progressive (10)
P4	54 (F)	3.4 × 3.0	5.3 × 3.1	cT2N3M1b IVb	AD	MAD	E19 del	NT	37%	35%	Gefitinib + RT	Progressive (14)	Progressive (14)
P5	73 (F)	2.6 × 1.7	4.3 × 2.8	cT2N0M1c IVb	AD	MAD	E19 del	NT	52%	52%	Gefitinib + RT	Progressive (18)	Stable (18)
P6	67 (M)	4.4 × 2.8	2.5 × 1.8	cT2N2M1b IVa	AD	MAD	E19 del	NT	26%	32%	Gefitinib + RT	Stable (5)	Stable (5)
P7	64 (F)	2.7 × 2.1	2.1 × 1.8	cT2N2M1b IVa	P-NSCC	MAD	L858R	NT	24%	37%	Gefitinib	Deceased (7)	Deceased (7)
P8	54 (F)	2.8 × 2.4	1.5 × 0.9	cT1N1M1b IVa	AD	MAD	L858R	NT	31%	34%	Gefitinib + CT + RT	Progressive (3)	Stable (3)
P9	61 (F)	3.2 × 2.8	2.5 × 2.4	cT2N3M1c IVb	AD	MAD	L858R	NT	60%	57%	Erlotinib + CT	Progressive (7)	Stable (7)
P10	50 (M)	1.7 × 0.6	1.9 × 1.7	cT1bN0M1c IVb	AD	MAD	–	L858R	24%	25%	Osimertinib	Stable (4)	Stable (4)
P11	66 (M)	1.5 × 1.3	2.6 × 1.3	cT1bN2M1c IVb	AD	MAD	–	L858R	94%	30%	Gefitinib	Progressive (17)	Stable (17)
P12	49 (M)	2.6 × 1.5	3.6 × 1.1	cT3N0M1c IVb	AD	MAD	G719X^b^	NT	47%	51%	Osimertinib + RT	Progressive (12)	Stable (12))
P13	59 (M)	5.6 × 6.3	1.2 × 1.4	cT3N0M1c IVb	AD	MAD	L861Q	NT	67%	79%	Gefitinib	Deceased (14)	Deceased (14)
P14	52 (M)	2.0 × 1.7	2.5 × 2.7	cT1N2M1b IVa	AD	MAD	EML4-ALK	NT	70%	33%	Crizotinib + RT	Stable (13)	Stable (13)
P15	63 (M)	3.4 × 3.7	7.4 × 7.9	cT2N2M1c IVb	AD	MAD	–	NT	47%	49%	CT	Progressive (5)	Progressive (5)
P16	53 (F)	4.2 × 7.4	2.1 × 2.3	cT4N0M1a IVb	AD	MAD	–	NT	35%	35%	CT + RT	Stable (3)	Stable (3)
P17	53 (M)	3.2 × 1.9	4.4 × 1.8	cT2N2M1b IVa	AD	MAD	–	NT	33%	74%	CT + RT	Deceased (2)	Deceased (2)

^a^Tumor purity was defined as the ratio of tumor to normal cells. ^b^ARMS-PCR cannot determine the precise amino acid change (X). *AD* Adenocarcinoma, *P-NSCC* Poorly differentiated non-small cell carcinoma, *MAD* Metastatic adenocarcinoma, *NT* Not tested, *CT* Chemotherapy, *RT* Radiotherapy ^c^Progressive disease, defined as > 20% increase in tumor size. Stable disease, defined as either no change or < 20% change in tumor size

### Tumor biopsy

Fine needle biopsies were taken as directed by the clinician from the primary lung tumor (PT) and the most accessible bone metastasis (BM). Biopsy tissue was washed twice in phosphate buffer saline (PBS), formalin fixed and paraffin-embedded (FFPE), and then sent for histology and genetic analysis.

### ARMS-PCR

For assessing immediate treatment options after diagnosis of LABM, primary tissue was analyzed by ARMS-PCR for common hotspot mutations. Rapid genetic profiling was performed using the Chinese FDA (cFDA) approved AmoyDx® *EGFR/ALK/ROS1* Mutations Detection Kit, a one-step real-time PCR test (combined reverse transcription and PCR amplification) designed for qualitative detection of 18 *EGFR* mutations (exons 18–21), 5 *ALK* gene fusions and 9 *ROS1* gene fusions.

### capSMART tumor analysis and identification of somatic variants

Comprehensive exome analysis of primary and metastasis FFPE biopsy tissue samples for somatic variants as well as matching blood samples for identifying background germline variants was performed by capture single molecule amplification and resequencing technology (capSMART) for a panel of 457 genes. Genes were selected mainly from the Catalogue of Somatic Mutations in Cancer (Forbes et al. 2015), the Cancer Genome Atlas (Weinstein et al. 2013), OncoKB (Chakravarty et al. 2017) and the Oncomine database (Rhodes et al. 2004). In brief, DNA extracted from FFPE tissue biopsies was fragmented to an average size of 300 bp, molecules were then end repaired and A-tailed and finally T tailed linkers were ligated on. The added linkers were a mix of 96 different molecular barcodes giving a high probability that each molecule was marked differently at both ends and thus uniquely barcoded. Libraries were amplified by PCR and resulting amplicons captured using biotinylated probes (120 nucleotides) for the 457 genes. Following elution, molecules were re-amplified using complementary sequencing primers and then paired end (PE) sequenced (2 × 150 bp) on the NovaSeq platform (Illumina).

Fastq sequencing reads were aligned to the hg19 reference genome using the Burrows Wheeler algorithm (Li and Durbin 2009). The resulting SAM files were converted to BAM file format and then sorted on genome coordinates using Samtools. To remove PCR bias (reads with the same molecular barcodes and same start and same stop positions), only the unique coded molecules were used for copy number analysis. After filtering out low mapping quality reads (MAQ < 20), the average depth of coverage (DoC) for each target was calculated using the GATK Depth Of Coverage algorithm (McKenna et al. 2010). After GC correction using LOESS regression method (Alkan et al. 2009), reads were normalized using the RPKM method (Chiang et al. 2019). For these steps, the tumor and matched normal sample was processed separately. Somatic SNVs and indels were finally identified by MutLoc (Berry Genomics in-house tools, unpublished), which maps the alternative base fraction compared to the hg19 reference genome.

### Copy number analysis of somatic variants

For copy number analysis of the normalized set of somatic variants, we calculated the log ratio of DoC for each target (tumor versus control), and then used the circular binary segmentation (CBS) algorithm to segment the log ratio profile into segments of equal copy number (Olshen et al. 2004). After segmentation, the CNV genes were extracted from the CBS segments. In brief, we first filtered out genes with less than 5 targets (target number ≤ 4). Then for each gene target, we calculated the segment value, which is the mean log ratio of all targets within this segment. If the segment value was ≥0.35, we marked this target as a target gain. If the segment value was ≤ − 0.5, we marked this target as a target loss. For any given gene, if the number of gain targets / all targets for this gene was ≥0.7, then this gene was marked as a gene amplification. If the number of loss targets / all targets for this gene ≥0.7, then this gene was marked as a gene deletion. Finally, for each amplification or deletion gene, we calculated its average log ratio using all of targets belonging to this gene and then calculated the average copy number.

For calculation of the mutation copy number n_*mut*,_ we used the following formula (Jamal-Hanjani et al. 2017):

$$ {n}_{mut}= VAF\frac{1}{p}\;\left[{pCN}_t+{CN}_n\;\left(1-p\right)\right] $$

where CN_t_ is the tumor locus specific copy number, CN_n_ is the normal locus specific copy number (assumed to be 2), *p* is the tumor purity calculated by Facets (Shen and Seshan 2016) and VAF is the variant allele frequency.

### Assessment of drugs for treatable genetic variants

Drugs for treatable genetic variants revealed by capSMART analysis of primary and secondary tumors were evaluated for their level of clinical utility for treatment of lung cancer using specific evidence codes documented in CIViC, OncoKB, Jax-CKB, CGI, MMatch and PMKB databases, following new evidence recommendations (Wagner et al. 2020). Level A (tier 1), evidence from professional guidelines or FDA approved therapies specific for a biomarker or disease; level B (tier 1), evidence from clinical trials with expert consensus; level C (tier 2), evidence from patient case studies and level D (tier 2), evidence from preclinical studies.

## Results

### Preliminary analysis of tumor biopsies

**Fig. 2:**
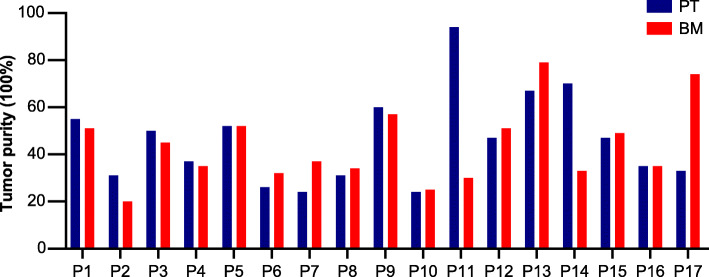
Tumor purity by histology. Purity was defined as the ratio of tumor to normal cells

Tumor assessments for the 17 patients (P) diagnosed with LABM are summarized in Table 1. All patients had stage IV disease and the histological subtype was adenocarcinoma (exception P7). With the exception of P11, P14 and P17, the tumor purity of the FFPE sections was relatively similar between matching primary tumor (PT) and bone metastases (BM) biopsies (Fig. 2). The range of tumor purities across the 34 biopsies varied from as low as 20% to as high as 95%.

**Fig. 3:**
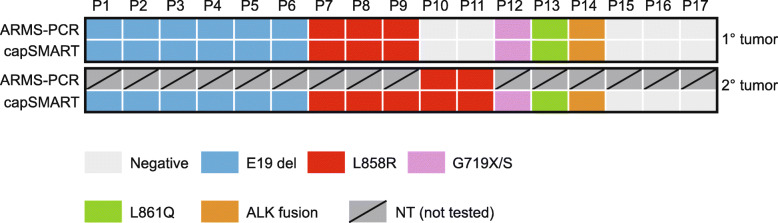
Concordance of ARMS-PCR and CapSMART for detection of *EGFR* and *ALK* fusion mutations

For rapid evaluation of targeted therapy options, PT and BM samples were initially screened for *EGFR* TK activity variants and *ALK*/*ROS* gene fusions (Table 1, Fig. 3). Thirteen patients (P1–13) were positive for *EGFR* mutations, including E19 deletions (*n* = 6), L858R (*n* = 5), G719X (*n* = 1) and L861 (*n* = 1) and, one patient (P14) was positive for an *EML4*-*ALK* gene fusion. The remaining three patients (P15–17) were mutation negative.

### Treatment outcomes for LABM patients

Based on disease severity, tumor assessments and genetic evaluation, the managing clinicians initiated personalized treatment regimens for each patient (Table 1, Supplementary Table 1). Disease was assessed by changes in the size of the biopsied PT and BM, after a post-treatment follow up period of 3–18 months. For the 13 *EGFR* mutation positive patients, a variety of TKIs such as Gefitinib, Erlotinib and Osimertinib (Cohen et al. 2004; Khozin et al. 2014; Popat 2018) were administered with or without chemotherapy or targeted radiation therapy. Disease was progressive in seven of these patients (P1, P3, P4, P5, P8, P9, P11 and P12) and stable in three others (P2, P6 and P10). However, two patients (P7 and P13) succumbed to the disease at 7 and 14 months post-treatment. P14 who was *ALK* fusion positive was administered the TKI Crizotinib (Peters et al. 2017) and after 13 months of treatment, disease was stabilized. For the three *EGFR*/*ALK* negative patients (P15, P16 and P17), standard chemotherapy and/or targeted radiation therapy regimens were administered. Disease was progressive in P15 and stable in P16 after treatment for 5 and 3 months, respectively. P17 succumbed to the disease 2 months post-treatment.

### Somatic variant profiling of primary tumor and secondary metastases

We applied the capSMART assay that surveys somatic exonic variants in 457 tumor-related genes (Supplementary Table 2A) and retrospectively re-evaluated the 17 archived PT and matching BM FFPE samples. QC analysis of the sequencing data (Supplementary Table 2B), showed high read coverage across all the 457 genes. In addition, by SNP analysis (Supplementary Table 2C), the SNP fingerprints of the PT, the BM and the germline genomic DNA for each patient were identical, indicating no sample mix ups. On this basis, the sequencing data derived allowed meaningful quantitative copy number analysis and thus the somatic SNVs, indels and CNVs patterns were directly comparable for each of the 17 PT and BM biopsy pairs.

**Fig. 4:**
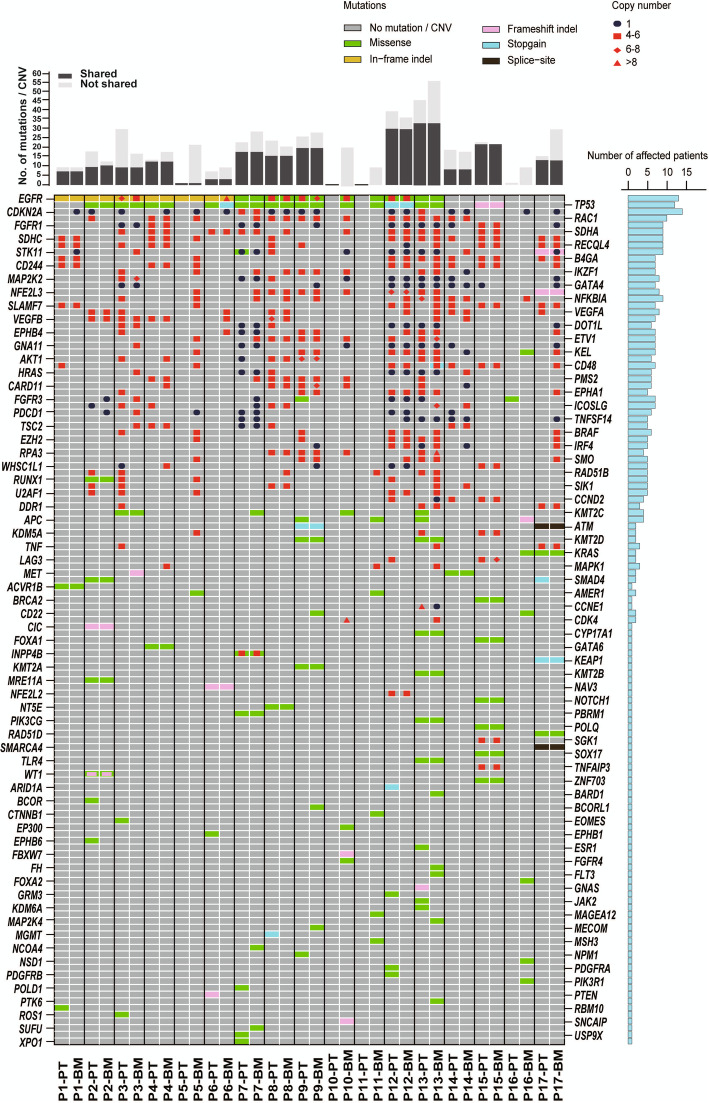
Genetic fingerprints of somatic variants in matching primary and secondary tumors. The different types of SNVs/indels and CNVs and associated copy number (CN) changes identified by capSMART analysis of patient tumors are indicated by color coding. PT = primary tumor; BM = bone metastasis. Shared SNVs/indels were scored when both PT and BM had a variant allelic frequency of ≥3%, shared gene amplifications were scored when both PT and BM had a CN ≥ 4 and shared gene deletions were scored when both PT and BM had a CN ≤ 1

All 17 patients displayed unique biopsy profiles involving various combinations of SNVs, indels and CNVs (Fig. 4). The most common SNVs/indels were associated with *EGFR* (13 patients) and *TP53* (12 patients). The remaining 97 variants in 78 genes were sporadically distributed in the tumor samples. The most common CNVs involved *EGFR* mutation amplifications (5 patients), followed by *TP53*, *CDKN2A*, *RAC1*, *FGFR1*, *SDHA*, *SDHC*, *RECQL4* and *STK11* deletions/duplications (> 8 of the 17 patients for each CNV). All the somatic *EGFR* variant types and the *EML4*-*ALK* gene fusion originally detected by ARMS-PCR were also confirmed by capSMART analysis (Fig. 2). Further, capSMART was able to additionally provide important copy number data for all the *EGFR* mutations and was also able to precisely identify the nature of *EML4*-*ALK* gene fusion (Supplementary Fig. S1).

Overall, for 14 patients (P1-P9, P12–15, P17), > 50% of the somatic variants identified (including the *EGFR* variants), were shared between the PT and BM. In the vast majority of cases, the copy numbers of the shared variants were also very similar. This suggests that in these patients, the secondary BM was most likely derived from a dominant clonal line present in the PT. The remaining non-shared variants presumably represent independent de novo passenger variants that subsequently arose by random events associated with ongoing tumorigenesis. In contrast, the PT and BM from patients P10, P11 and P17 showed very few somatic variants, and of these, none were shared genetic variants. For patients P10 and P11, the *EGFR* driver mutation L858 was found exclusively in the secondary BM by both ARMS-PCR and capSMART analysis. While low levels of *EGFR* mutations cannot be completely excluded in the PT, the divergent genetic profiles suggest that the BMs most likely originated from a less dominant L858 positive clonal line present in the PT.

### Identification and evaluation of alternative therapeutic targets for LABM patients

From the complex somatic variant profiles of the matching PT and BM biopsies established for the 457 genes tested (Fig. 4), we re-assessed the new information to determine whether there were any other potential treatable mutations which could have been administered as an alternative to the original therapies (Table 2). In regard to the 13 *EGFR* positive patients P1, P2, P4 and P5, P7 and P13 (both tumors) and P10 and P11 (only BM), all had low level *EGFR* mutation amplifcations (< 5 copies) and thus no additional treatable drug was indicated. In contrast, for patients P3 P9, P12 and P13 (both tumors) and P6 (only BM), all had clinically significant *EGFR* mutation amplifications (> 5 copies). Thus, these patients may have benefited from the level B drug Erlotinib which is known to be more effective than Gefitinib for *EGFR* driver amplifications (Tsao et al. 2005).

**Table 2:** Treatable genetic variants and level of clinical utility

Patient	Gene	Tumor genetic variants (copy number)		Treatment options	
		1° tumor	2° tumor	Drug^a^	Level of clinical utility
P1	*EGFR*	E746_A750delELREA (1)	E746_A750delELREA (1)	Gefitinib	Level A
P2	*EGFR*	E746_A750delELREA (1)	E746_A750delELREA (2)	Gefitinib	Level A
P3	*EGFR*	E746_A750delELREA (6)	E746_A750delELREA (5)	Gefitinib	Level A
		Amplification	Amplification	**Erlotinib**	Level B
P4	*EGFR*	L747_A750delinsP (1)	L747_A750delinsP (1)	Gefitinib	Level A
P5	*EGFR*	E746_A750delELREA (0.1)	E746_A750delELREA (2)	Gefitinib	Level A
P6	*EGFR*	E746_A750delELREA (1)	E746_A750delELREA (9)	Gefitinib	Level A
		No amplification	Amplification	**Erlotinib**	Level B
	*TP53*	Negative	Q331X (2)	**AZD1775**	Level D
P7	*EGFR*	L858R (2)	L858R (3)	Gefitinib	Level A
P8	*EGFR*	L858R (5)	L858R (4)	Gefitinib	Level A
P9	*EGFR*	L858R (5)	L858R (6)	Erlotinib	Level A
		Amplification	Amplification	Erlotinib	Level B
	*ATM*	W3052X (2)	W3052X (2)	**Olaparib**	Level D
P10	*EGFR*	Negative	L858R (4)	Osimertinib	Level A
	*CDK4*	No amplification	Amplification (17)	**Abemaciclib**	Level B
P11	*EGFR*	Negative	L858R (2)	Gefitinib	Level A
P12	*EGFR*	G719S (5)	G719S (5)	Osimertinib	Level B
		Amplification	Amplification	**Erlotinib**	Level B
	*TP53*	R196X (2)	R196X (2)	**AZD1775**	Level D
P13	*EGFR*	L861Q (2)	L861Q (3)	Gefitinib	Level A
	*TP53*	R248W (2)	R248W (2)	**AZD1775**	Level D
P14	*ALK*	Fusion (0.3)	Fusion (0.2)	Crizotinib	Level A
P15	*TP53*	F134Pfs (1)	F134Pfs (1)	**AZD1775**	Level D
	*FGFR1*	Amplification (5)	Amplification (6)	**Dovitinib**	Level D
P16	*FGFR3*	R248C (0.1)	Negative	**Dovitinib**	Level D
	*KRAS*	Negative	G12D (1)	**Cobimetinib**	Level D
P17	*KRAS*	G12C (1)	G12C (1)	**AMG-510**	Level B

^**a**^Drugs shown in normal text represent the initial therapy based on ARMS-PCR genetic test. Drugs shown in bold text represent potential alternative therapies revealed by capSMART test. Amplification of gene variants or normal gene sequences was defined by a copy number of > 5

Apart from *EGFR* amplifcation mutations, P12 and P13 had additional *TP53* mutations p.R196X and p.R248W in both tumors which are potentially treatable with the level D drug AZD1775 (Alexandrova et al. 2015). In contrast, P6 had the *TP53* mutation p.Q331X exclusively in the BM biopsy which is also potentially treatable by AZD1775 (Richer et al. 2017). Moreover, P9 had the *ATM* mutation p.W3052X in both the PT and BM biopsies, potentially treatable by the level D drug Olaparib (Mateo et al. 2015). Lastly, P10 had a *CDK4* gene amplification exclusively in the BM biopsy which is treatable by the level B non lung cancer drug Abelaciclib (Dickson et al. 2013).

Analysis of the mutation profiles for P14 who was positive for an *EML4*-*ALK* gene fusion in both PT and BM biopsies, revealed no additional drug options other than the original drug Crizotinib (Peters et al. 2017). For the 3 *EGFR/ALK* negative patients P15, P16 and P17, capSMART analysis did reveal new drug therapy options. Firstly, both the PT and BM tumors of P15 had *FGFR1* gene amplifications that can be targeted by the level D drug Dovitinib (Lim et al. 2016). Secondly, P17 had the *KRAS* mutation p.G12C in both tumors treatable with the level B drug AMG-510 (Canon et al. 2019). Lastly, P16 had the *FGFR3* mutation p.R248C exclusively in the PT biopsy and the *KRAS* p.G12D mutation exclusively in the BM biopsy and, both mutations are targetable with the level D drug Dovitinib and the level D drug Cobimetinib (Lieu et al. 2017), respectively.

## Discussion

In this study we retrospectively reanalyzed the original PT and BM biopsies of a small cohort of newly diagnosed LABM patients using the capSMART method that surveyed somatic mutations and CNVs in a panel of 457 tumor associated genes. This approach allowed us to firstly examine the naïve genetic profile of the 457 tumor related genes and the clinically significant genetic changes that had evolved in the tumors before treatment and, secondly to compare the genetic relatedness between the paired PT and secondary BM biopsies. Moreover, from detailed analysis of the tumor gene mutation and CNV profiles defined for each patient, we were able demonstrate that our molecular test could reveal additional treatable mutation(s) above and beyond those initially identified by the simple ARMS-PCR test.

Amongst the 17 patients, apart from *EGFR*, the genetic PT and BM profiles of somatic variants identified in other affected genes were very different, indicating divergent clonal evolution in each patient which is a common feature of LA as well as many other cancers (Jamal-Hanjani et al. 2017). However, in the pretreatment phase, there was a high level of genetic relatedness between the paired PT and BM biopsies for 15 of the 17 patients. The exceptions were P10 and P11 whereby only the *EGFR* L858R mutation was detected in the BM biopsy. We propose in these two cases that there was intratumor heterogeneity in the PT and that the BMs were formed from a different clonal line in the PT. In support of this notion, from a large study of Chinese patients with advanced non-small cell lung adenocarcinoma, genetic analysis of biopsies taken at multiple sites in the PT showed significant heterogeneity for treatable mutations in 30% of patients (Bai et al. 2013).

Our general findings between paired PT and BM samples from LABM patients are consistent with the genetic analyses of other cancer types, whereby the majority of genetic changes found in the PT are also found in the secondary BMs, although BMs do have a tendency to genetically diverge due to ongoing genomic instability. There is a growing body of data that suggests that the secondary BMs which form in some cancer patients are usually seeded by the main clonal line from the PT (Hu et al. 2019; Ramaswamy et al. 2003; Reiter et al. 2018; Sottoriva et al. 2015). Whether seeding of BMs by rogue clones occurs early in the development of the PT or at a more advanced stage is yet to be resolved, but both mechanisms may be operative in some patients. Nonetheless, from the study of this small cohort of patients, it is clear that treatment options for LABM applicable to the PT may also be useful to help control the progression of existing genetically related BMs as well as the spread of rogue clonal lines which have high intrinsic potential to develop into new BMs once seeded in the bone, liver or brain tissues.

Using the simple ARMS-PCR test, 14 of the 17 newly diagnosed patients were initially treated with TKIs for either *EGFR* or *ALK* fusion mutations that were present in the PT and the BM biopsies. Thus, the first therapy TKI option selected by the clinician could have been potentially effective against both the PT and the BMs. However, we observed that post treatment, 8 of the 14 patients still had progressive disease while 4 had stable disease and 2 patients had unfortunately succumbed to the disease. In the majority of cases the ongoing survival rates were significantly longer with directed TKI treatment compared to LABM patients undertaking conventional treatments (Bender 2014). It is interesting to speculate that if the more comprehensive genetic profile we obtained by capSMART was available at the time of diagnosis of LABM, as to whether a more effective regime based on TKI inhibitors could have been administered. Certainly, alternative and additional drug therapy options of level A, B and D clinical utility were revealed for nine of the 17 LABM patients, including six* EGFR* positive and three *EGFR* negative patients. Further, in 4 LABM patients, treatable mutations were found exclusively in the secondary BM, presenting additional options to specifically treat the BM(s) in these patients.

Our capSMART test also enabled quantitative assessment of the copy numbers of treatable mutations and deletions/duplications within the PT and the BM tissues which could be an aid to gauge the clinical significance of these variants. Thus for clinicians managing these LABM patients, it could have been possible to use this information to design and tailor a more personalized TKI treatment regimen with or without chemotherapy or radiotherapy that potentially would have been more effective in targeting both the PT and BM tissues harboring these tumorigenic mutations. On this basis, further studies in a larger cohort of newly diagnosed LABM patients may assist to apply the knowledge gained from using a more comprehensive gene test and determine whether the disease can be more effectively stabilized, and importantly, extend survival rates above current median values.

While the main strength of this study was access to PT and secondary BM biopsy samples from a valuable set of patients with LABM, there were some clinical and technical limitations. Firstly, tumor analyses were restricted to biopsy samples from the PT and the most accessible secondary BM. Thus, due to clinical risk, we were unable to obtain biopsy samples from other secondary tumors that had additionally formed in some of the patients. Secondly, we did not collect biopsies from multiple tumor sites to assess intratumor heterogeneity. Such an approach may have revealed additional treatable somatic variants against other clonal cell lines, particularly for P10 and P11 where there was strong evidence of discordant genetic profiles between the PT and the secondary BM. Thirdly, while genetic profiles were focused on a preselected panel of 457 genes, a more comprehensive whole exome sequencing approach may have yielded additional treatable somatic variants in other tumor related genes.

## Conclusion

LABM is an aggressive disease whereby effective treatment needs to be rapidly administered to slow disease progression and improve survival rates. We suggest that based on our findings, the new standard of care should involve an initial comprehensive screen of the PT biopsy together with any available BMs that are accessible for biopsy. This approach will ensure that the treating clinician is provided with more personalized genetic information to tailor effective targeted therapy options and develop a more effective treatment regimen with or without chemo or radiation therapy. Further, with the advent of liquid biopsy as a means to survey the ctDNA released from clonal variants of PT and BM tissues (Cohen et al. 2018; Phallen et al. 2017), including tumors that are accessible or non-accessible by invasive biopsy, it will be possible to apply a screening technique like capSMART to provide a more complete picture of the clinically-significant somatic tumor variants and thus provide options for alternative therapy choices, particularly if the first choice regimen proves to be ineffective.

## Supplementary information

**Additional file 1.**

**Additional file 2.**

**Additional file 3.**

## Data Availability

The datasets used and/or analyzed during the current study are available from the corresponding author on reasonable request.
