# Supplementary material for: Genetic profiling of primary and secondary tumors from patients with lung adenocarcinoma and bone metastases reveals targeted therapy options
Source: Mol Med. 2020 Sep 17;26:88. doi: 10.1186/s10020-020-00197-9 (PMC7499871; doi:10.1186/s10020-020-00197-9)
Supplement: Supplementary file 3 — Additional file 3. [file 10020_2020_197_MOESM3_ESM.pdf]

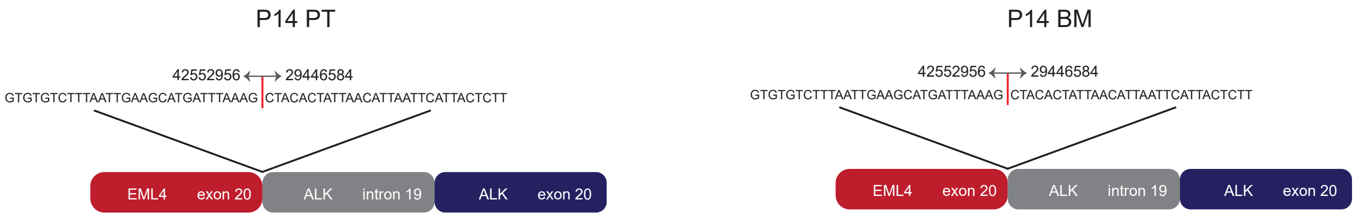

P14 PT
42552956
29446584
GTGTGTCTTTAATTGAAGCATGATTTAAAG
CTACACTATTAACATTAATTCATTACTCTT
EML4 exon 20
ALK intron 19
ALK exon 20
P14 BM
42552956
29446584
GTGTGTCTTTAATTGAAGCATGATTTAAAG
CTACACTATTAACATTAATTCATTACTCTT
EML4 exon 20
ALK intron 19
ALK exon 20
